# Erythrocyte long-chain omega-3 fatty acid levels are inversely associated with mortality and with incident cardiovascular disease: The Framingham Heart Study

**DOI:** 10.1016/j.jacl.2018.02.010

**Published:** 2018-03-02

**Authors:** William S. Harris, Nathan L. Tintle, Mark R. Etherton, Ramachandran S. Vasan

**Affiliations:** Department of Internal Medicine, Sanford School of Medicine, University of South Dakota; OmegaQuant Analytics, LLC, Sioux Falls, SD, USA; Department of Mathematics & Statistics, Dordt College, Sioux Center, IA, USA; Department of Neurology, Massachusetts General Hospital, Harvard Medical School, Boston, MA, USA; National Heart Lung and Blood Institute’s, Boston University’s Framingham Heart Study, Framingham, MA, USA; Departments of Cardiology and Preventive Medicine, Department of Medicine, Boston University School of Medicine, Boston, MA, USA; Department of Biostatistics, Boston University School of Public Health, Boston, MA, USA

**Keywords:** Epidemiology, Prospective cohort study, Eicosapentaenoic acid, Docosahexaenoic acid, Omega-3 fatty acids

## Abstract

**BACKGROUND:**

The extent to which omega-3 fatty acid status is related to risk for death from any cause and for incident cardiovascular disease (CVD) remains controversial.

**OBJECTIVE:**

To examine these associations in the Framingham Heart Study.

**DESIGN:**

Prospective and observational.

**SETTING:**

Framingham Heart Study Offspring cohort.

**MEASUREMENTS:**

The exposure marker was red blood cell levels of eicosapentaenoic and docosahexaenoic acids (the Omega-3 Index) measured at baseline. Outcomes included mortality (total, CVD, cancer, and other) and total CVD events in participants free of CVD at baseline. Follow-up was for a median of 7.3 years. Cox proportional hazards models were adjusted for 18 variables (demographic, clinical status, therapeutic, and CVD risk factors).

**RESULTS:**

Among the 2500 participants (mean age 66 years, 54% women), there were 350 deaths (58 from CVD, 146 from cancer, 128 from other known causes, and 18 from unknown causes). There were 245 CVD events. In multivariable-adjusted analyses, a higher Omega-3 Index was associated with significantly lower risks (*P*-values for trends across quintiles) for total mortality (*P* = .02), for non-CVD and non-cancer mortality (*P* = .009), and for total CVD events (*P* = .008). Those in the highest (>6.8%) compared to those in the lowest Omega-3 Index quintiles (<4.2%) had a 34% lower risk for death from any cause and 39% lower risk for incident CVD. These associations were generally stronger for docosahexaenoic acid than for eicosapentaenoic acid. When total cholesterol was compared with the Omega-3 Index in the same models, the latter was significantly related with these outcomes, but the former was not.

**LIMITATIONS:**

Relatively short follow-up time and one-time exposure assessment.

**CONCLUSIONS:**

A higher Omega-3 Index was associated with reduced risk of both CVD and allcause mortality.

## Introduction

Several recent studies have linked higher blood levels and/or dietary intakes of the long-chain n-3 polyunsaturated fatty acids (PUFAs) with greater longevity. Plasma phospholipid n-3 PUFA levels were inversely associated with total mortality rates in the Cardiovascular Health Study,^1^ and similar associations were seen for this endpoint with the red blood cell (RBC) content of eicosapentaenoic acid (EPA) plus docosahexaenoic acid (DHA) in the Heart and Soul Study.^2^ This latter metric, called for simplicity the Omega-3 Index, has been proposed as a risk factor for death from cardiovascular disease (CVD).^3,4^ Consistent with these observations, there is an inverse relationship between the Omega-3 Index and the rate of telomere attrition, a marker of cellular aging.^5^ Although early randomized controlled trials with n-3 PUFAs found reduced overall mortality,^6,7^ other more recent studies^8–11^ have not confirmed such a protective effect. Some of the potential reasons why recent studies may have yielded null results for intervention with n-3 PUFAs (background use of statins,^12^ short follow-up periods, low n-3 PUFA doses, improvements in acute care, etc.) have been reviewed.^13,14^ In the present investigation, we examined the relations between RBC n-3 PUFA levels in participants in the Framingham Heart Study’s (FHS’s) Offspring cohort and total mortality as the primary endpoint, with secondary endpoints of death from CVD and other causes, and of incident coronary heart disease (CHD), CVD, and ischemic stroke.

## Methods

The FHS is a longitudinal community-based cohort study that was initiated in 1948. Adult children of the original cohort were recruited in 1971 into the Framingham Offspring Cohort. The selection criteria for this cohort (and for the more racially diverse Framingham Omni Cohort) have previously been described.^15,16^ We evaluated Framingham Offspring/Omni participants (n = 3021) who attended their 8th/3rd examination cycles (2005–2008). Participants were excluded in hierarchical order if they were missing RBC FA measurements or relevant clinical covariates (n = 122), leaving 2899. We also excluded 399 with a history of CVD (ie, nonfatal CHD or stroke), leaving 2500 for the present investigation. The study protocol was approved by the Institutional Review Board of the Boston University Medical Center. Informed consent was provided by all participants.

## Covariates and mortality outcomes

We considered 18 primary baseline demographic and CV risk covariates: sex, age, body mass index, marital status, education level, employment status, health insurance status, regular aspirin user, prevalent hypertensive status, use of cholesterol-lowering drugs, prevalent diabetes, history of CVD, alcohol consumption, smoking status, physical activity (in metabolic equivalent units), the total cholesterol to high-density lipoprotein cholesterol ratio, systolic blood pressure, and C-reactive protein. Four mortality endpoints were examined: total, CVD [fatal myocardial infarction (MI), CHD death, sudden cardiac death, fatal ischemic stroke, or other CVD death), cancer, and other (ie, non-CVD, non-cancer; Note that in the Framingham Heart Study, “other” causes of death were not specifically identified]. Incident CVD-related endpoints were also examined: total CVD (total stroke, total CHD, or CVD mortality), total stroke (any fatal or nonfatal ischemic stroke), and total CHD (fatal or nonfatal MI, CHD death or sudden cardiac death).

## RBC FA analysis

Blood was drawn after a 10- to 12-hour fast into an EDTA tube, and RBCs were separated from plasma by centrifugation. The RBC fraction was frozen at −80°C immediately after collection. RBC FA composition was determined as described previously.^17^ Briefly, RBCs were incubated at 100°C using boron trifluoride methanol and hexane to generate FA methyl esters that were then analyzed by gas chromatography with flame ionization detection. N-3 FAs analyzed included α-linolenic acid (ALA; 18:3n3), EPA (20:5n3), docosapentaenoic acid (DPA; 22:5n3), DHA (22:6n3), and the Omega-3 Index (EPA + DHA).^3^ The coefficients of variation for these were 10.7%, 7.8%, 3.8%, 3.3%, and 2.4%, respectively.

## Statistical analysis

Sample characteristics were summarized using standard statistical metrics (eg, means, standard deviations (SDs), and correlations). Hazard ratios were estimated using the survival package in R.^18^ Primary analyses related incident clinical outcomes by date of event (or censoring) to quintiles of the Omega-3 Index, with follow-up analyses adjusting for demographic and medical history covariates. Secondary analyses explored relationships of outcomes by quintiles of individual n-3 PUFA levels, of a modified Omega-3 Index that included DPA, and by risk category of the Omega-3 Index (high risk, ≤4%; intermediate risk, >4% to <8%; and low risk, ≥8%).^19^ In other analyses, we included the n-6 FAs as covariates and also compared the n-6:n-3 ratio with the Omega-3 Index. Finally, baseline total cholesterol levels were compared with the Omega-3 Index in the same models to get a sense of how this emerging risk factor compared with an established one. All analyses used 2-sided tests at the *P* < .05 statistical significance level.

## Results

### Cohort description

This analysis was performed on 2500 Framingham Offspring study participants who were free of CVD at baseline and for whom FAs, clinical outcomes, and demographic covariates were available. Follow-up ended on December 31, 2016 (maximum, 11.2 years; median, 7.3 years). Table 1 provides the baseline characteristics of the cohort by mortality status. The average age was 66 years, and there were more women (57%) than men (43%). There were 350 deaths during the follow-up period: 58 were attributable to CVD (17%), 146 to cancer (42%), 128 to other known causes (37%), and 18 from unknown causes (5%). Overall, there were 245 incident cases of CVD, 119 of CHD, and 105 ischemic strokes. (Case and control numbers and length of follow-up for each outcome are in Table S1).

### The Omega-3 Index and total and cause-specific mortality

**Figure 1:**
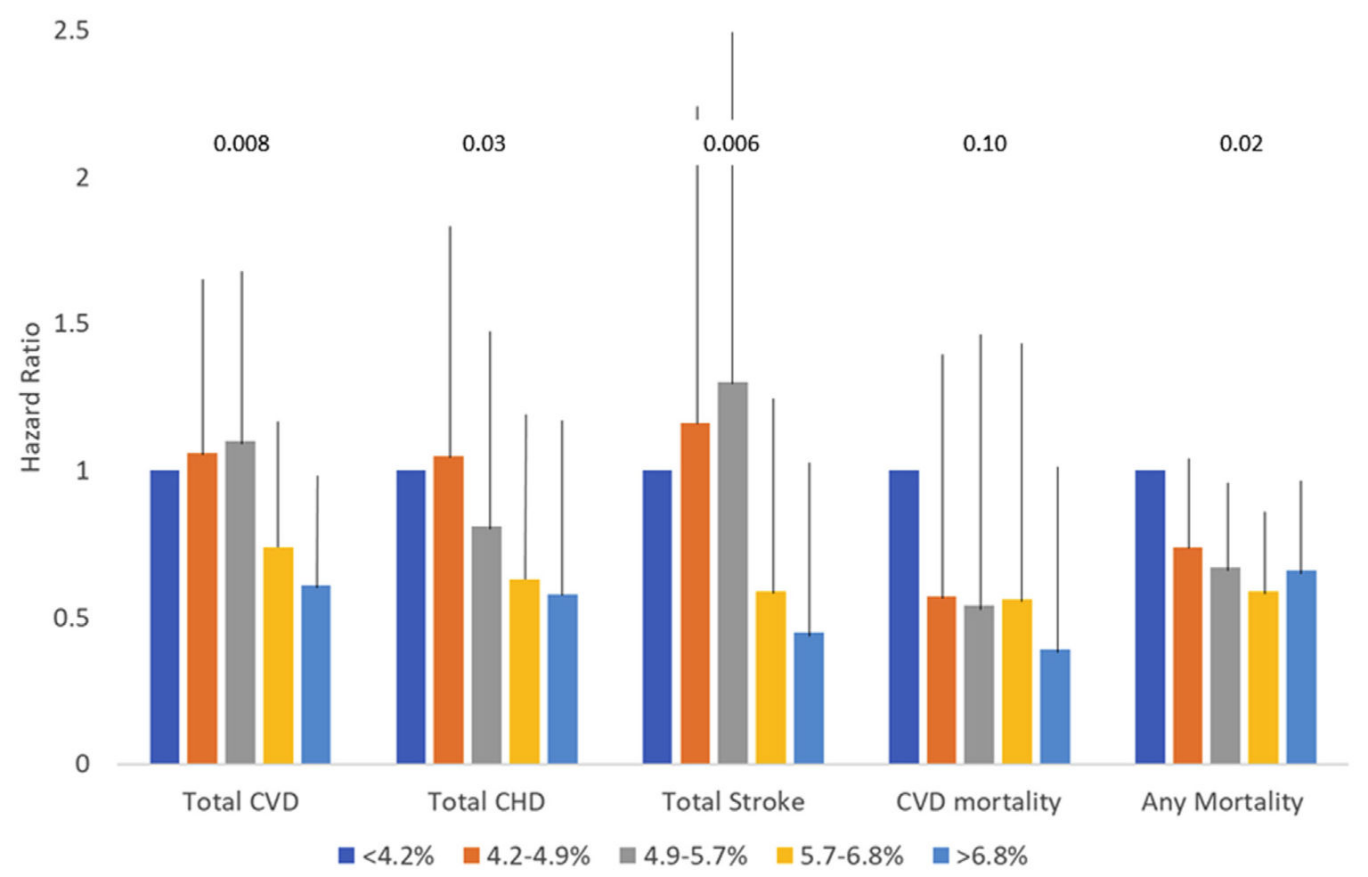
Relations between quintiles of the Omega-3 Index and hazard ratios (+95% confidence interval) for death from CVD (n = 58), cancer (n = 146), other causes (n = 128), and all causes (n = 350, including 18 of unknown causes). Data are from 2500 participants free of baseline CVD followed for a median of 7.3 years. Adjusted for all variables in Table 1. *P*-values for trend are shown above the columns. CVD, cardiovascular disease; CHD, coronary heart disease.

We estimated the associations between adjusted risk for fatal outcomes across quintiles of the Omega-3 Index (Fig. 1, with details in Table S2). In the multivariable-adjusted model, death from any cause was significantly and inversely associated with the Omega-3 Index (*P* for trend = .02). When comparing individuals in the lowest quintile of the Omega-3 Index (<4.2%) to those in the highest (>6.8%), risk was 34% lower. Median values for the extreme quintiles were 3.7% and 7.8%. The modified Omega-3 Index (which included DPA) performed no differently from the original metric (Table S3), and an examination of the relations between risk and the three Omega-3 Index risk categories found a significant trend across the categories (*P* = .04), but only the intermediate category differed significantly from the high-risk category (possibly because there were 10-fold more subjects in the intermediate than the highest categories; Table S4). When we controlled for RBC n-6 PUFA levels in the original analysis, or substituted the n-6:n-3 ratio for the Omega-3 Index, there was no material improvement in mortality hazard ratios (Table S5). In neither case did the n-6 PUFAs alter the n-3 PUFA associations.

Regarding cause-specific mortality, the Omega-3 Index was only significantly associated with risk for death from “other” (non-CVD, non-cancer) causes (*P* for trend = .009), with the participants in the first (reference) quintile being at significantly higher risk than those in quintiles 2–5. There was a trend for reduced risk for CVD across Omega-3 Index levels (eg, there was a 61% lower risk between extreme quintiles), but with wide confidence intervals, the differences were nonsignificant. The results for total and cause-specific mortality were similar in analyses including all 2899 participants (ie, including those with a history of CVD; Table S6).

### The Omega-3 Index and CVD outcomes

The Omega-3 Index was significantly and inversely associated with total CVD, total CHD, and total stroke after adjusting for covariates in a linear trend analysis across quintiles (Table 2; *P* values for trends = .008, .03, and .006, respectively). Unadjusted hazard ratios are shown in Table S7. The hazard ratio for total CVD was significantly reduced in quintile 5 vs 1, but for total CHD or total stroke, no individual quintile differed significantly from the reference. Similar findings were observed in the full cohort (n = 2899) for total CHD and total stroke, but the relationship with total CVD was no longer statistically significant (Table S8). Using the modified Omega-3 Index (with DPA) did not change the pattern of results (Table S3), and when Omega-3 risk categories were tested, the general pattern of relationships remained the same, but they were mostly nonsignificant (Table S4). Finally, neither including n-6 PUFAs as covariates nor replacing the Omega-3 Index with the n-6:n-3 ratio altered the original results (Table S5).

### Individual RBC n-3 PUFAs and outcomes

The adjusted risk for experiencing the endpoints of interest by each of the four n-3 PUFAs separately is shown in Table 3 (unadjusted hazard ratios in Table S9). The plant-derived n-3 PUFA, ALA, was not significantly associated with reduced risk for any of the outcomes tested (Table 3). In general, as the levels of the EPA and (especially) DHA increased, risk for disease outcomes and total (and other) mortality decreased (Table 3). The only outcome that DPA was (marginally, *P* = .059) associated with was death from other causes (Table 3).

### Omega-3 Index vs total cholesterol

In the head-to-head comparison of the Omega-3 Index and total cholesterol for total and CVD mortality and for the 3 CVD outcomes, the former metric was significantly associated with risk for 4 of the 5 outcomes, whereas the total cholesterol level, in the same models, was not associated with risk for any of these outcomes (Table 4).

### Discussion

In this study of participants in the Framingham Offspring study, the baseline Omega-3 Index at about 66 years of age was inversely associated with total mortality over the next 7 years—with values of the Omega-3 Index significantly lower in those who died during follow-up than those who did not. The same was generally true for the 2 components of the Index, EPA and DHA, but not for RBC DPA n-3 or ALA. Findings for the Omega-3 Index persisted in a model adjusted for 18 other relevant variables including plasma lipids and C-reactive protein.

We recently reported similar findings to these in the Women’s Health Initiative Memory Study where there was a significant inverse trend across quartiles of the Omega-3 Index for total mortality, with the highest quartile being at 22% lower risk for death compared with the lowest.^20^ That study followed a nationwide sample of 6500, approximately 70-year-old women for 15 years. Kleber et al. also found a 9% decrease in risk for all-cause mortality per 1 SD increase in the Omega-3 Index after 10 years of follow-up in 3259 German CHD patients.^21^ In the present study, we followed up 2500 men and women for half as long and found an interquintile risk reduction of 34%. We also found reduced risk for total CVD events, CHD events, and stroke. The approximately 50% lower risk for CVD mortality was, however, not statistically significant, most likely due to having only 58 events in the analysis (see below). Nevertheless, these findings are generally consistent with those of several meta-analyses that all linked higher n-3 PUFA biomarker levels with reduced risk for “coronary events”^22^ or fatal CHD.^19,23^ This study was somewhat unique, however, in exploring relations between RBC n-3 PUFA levels instead of whole plasma or plasma phospholipid n-3 PUFAs. But since these markers of n-3 PUFA status are strongly intercorrelated,^24^ this coherence is expected.

The causes of death most strongly associated with the Omega-3 Index were non-CVD and non-cancer, ie, “other” causes. By design, the FHS focused primarily on CVD outcomes, so details of the other causes of death are unavailable. An 11% reduction (per 1 SD increase in the Omega-3 Index) in risk for death from other causes was also seen in the women’s study mentioned previously.^20^ It is unclear what mechanism might explain this observation; but since n-3 PUFAs are known to have a wide variety of effects on cell membrane biology,^25,26^ a systemic impact of higher levels of these FAs on overall cellular health is possible. As noted earlier, several recent randomized trials of fish oil with total mortality as an endpoint have been null, but they typically showed trends toward a benefit.^27,28^ More consistent with our observations was a 2017 meta-analysis,^29^ which found a significant 19% reduction in risk for coronary death associated with fish oil treatment. Two nonrandomized retrospective studies using governmental databases to track patient outcomes (one from England^30^ and another from Italy^31^) reported lower risk for all-cause mortality in those CHD patients who had been prescribed 1 g of fish oil at discharge. Finally, higher plasma long-chain n-3 FA levels were strongly associated with reduced risk for total mortality and CHD outcomes in renal transplant patients in Norway.^32^

A somewhat surprising observation in this study was the low rate of CVD in this population, with only 17% of deaths being attributable to CVD (as opposed to 42% from cancer). Part of this is clearly the exclusion from the analysis set of participants with CVD at baseline, but even including these individuals, the CVD death rate was only 22%. This relatively low CVD death rate should perhaps not be surprising given the reduction in heart disease mortality in the United States over the last several decades. For example, the age-standardized death rate from heart disease per 100,000 people dropped from 520 in 1969 to 167 in 2014, a 68% decline.^33^

With regard to the individual RBC n-3 PUFAs studied here, in the multivariable-adjusted model, ALA was not significantly related to any measured outcome in the quintile analysis. The null mortality findings for ALA agree with some previous studies,^34,35^ but higher circulating ALA levels (primarily plasma and plasma phospholipids) have been favorably associated with CVD death,^23^ and ALA intake was associated with reduced total mortality in a large randomized clinical trial (RCT) from Spain.^36^ So, evidence for ALA remains mixed. Turning to DPA, in the final model, it was not related with any examined outcome (although trends were favorable). This contrasts with a previous meta-analysis in which this FA (again, more often measured in whole plasma or plasma phospholipids) was inversely associated with risk for CVD death.^23^ In light of this and other prior reports (eg, in heart failure^37^), the utility of RBC DPA as a risk marker remains unclear. Nevertheless, because of the growing evidence for a beneficial role of DPA,^38^ it is reasonable to ask why this FA is not a component of the Omega-3 Index. Including it would be reasonable if adding it to EPA and DHA improved the utility of the metric in risk assessment. In the present study, we compared the original and the modified Omega-3 Index (Table S3) and found essentially the same associations with outcomes for both. This would be expected since the correlation between these 2 predictor variables is 0.98. Hence, our findings provide no compelling reason to alter the original definition/components of the Omega-3 Index.

We found that the risk of stroke was inversely associated with the Omega-3 Index, but not in a linear manner, ie, hazard ratios were reduced only in the upper 2 quintiles; however, the trend across quintiles was significant. Risk reduction was largely associated with DHA, where risk was 59% lower in the fifth (vs the first) quintile (*P* < .05). An association between serum long-chain n-3 PUFA levels and stroke risk has been reported previously in cross-sectional studies.^39,40^ The impact of marine n-3 PUFA levels on ischemic stroke may extend beyond the risk of incident event. Lower plasma proportions of EPA and DHA have been shown to be independently associated with increased ischemic stroke severity and, for DHA, poor functional outcomes at 90 days after stroke.^41^ In addition, a higher ratio of EPA or DHA to arachidonic acid in serum was associated with lower likelihood of early neurologic deterioration after ischemic stroke.^42^ A 2012 meta-analysis of 32 observational cohorts and 13 n-3 RCTs reported inverse associations with stroke and fish intake, but no significant relations with either circulating biomarkers or with supplementation,^43^ although a 2017 study of plasma phospholipid omega-3 FA and incident stroke from 3 cohorts found inverse relations with DHA as we did here.^44^ A randomized, open-label study in patients surviving myocardial infarction^45^ showed a reduction in risk of nonfatal stroke with n-3 FA supplementation, and an open-label study in Japan demonstrated no effect on primary prevention of stroke but a 20% relative reduction in recurrent total stroke with EPA supplementation.^46^ However, some studies have found no relations between n-3 PUFA levels and stroke,^47,48^ so the n-3 PUFA and stroke literature is mixed, and more studies are needed.

A variety of exploratory analyses were conducted here. Given the controversies regarding the CVD effects of the n-6 PUFAs and the frequent use of the n-6:n-3 ratio, in one analysis we controlled for RBC n-6 PUFA levels and in another we directly compared the ratio with the Omega-3 Index. In neither case did the n-6 PUFAs add or detract from the omega-3 effect. Hence, this study found no support for using a combined metric with n-6 and n-3 PUFAs. Finally, we compared associations with disease outcomes for the Omega-3 Index with that of total cholesterol, one of the most well-known CVD risk factors. This analysis was undertaken to give some perspective of the relative sensitivity of these 2 biomarkers. In head-to-head comparisons with the same multivariable models, a higher Omega-3 Index was significantly associated with reduced risk for 4 of the 5 outcomes studies, but cholesterol was unrelated to risk for any outcome. This may be because about 38% of participants were on a cholesterol-lowering medication, and so cholesterol levels at baseline were therapeutically reduced; because 399 subjects with a diagnosis of CVD at baseline were excluded; or because those who were particularly susceptible to cholesterol-induced CHD may have already died. There is indeed some controversy regarding the predictive power of serum cholesterol in older individuals.^49^ Nevertheless, in the unique context of this study in which we controlled for 17 other variables, cholesterol levels were unassociated with these outcomes. Whatever the reasons, this observation supports the need for further research to more clearly define the relative roles of traditional and emerging biomarkers in risk stratification.

The originally proposed (in 2004) clinical cut-points for the Omega-3 Index were <4% (high risk) and >8% (low risk).^3^ Although these have recently been confirmed in a meta-analysis including 10 cohorts and over 27,000 subjects,^19^ it was of interest to explore their utility in the present study as well. We found a significant trend across risk categories for total mortality and significant reductions in risk for the intermediate group compared with the high group, but not for the >8% category. This may reflect a nonlinear relationship between the Omega-3 Index and risk as has been suggested by others,^25^ or the fact that only 10% of the sample was in the >08% category. Nevertheless, the hazard ratio of 0.69 for any death in the >8% category is at least suggestive of lower risk.

As alluded to earlier, mechanisms that may explain the associations between higher RBC n-3 PUFA and improved longevity and reduced CVD risk are not clearly understood, but there are beneficial effects of these FAs on a variety of CV risk factors. These include reductions in serum triglyceride levels,^50^ blood pressure,^51^ platelet aggregation,^52^ heart rate,^53^ susceptibility to ventricular fibrillation (in some settings),^54^ inflammatory markers,^55,56^ and plaque vulnerability^57,58^ along with improvements in endothelial function.^59^ The observation that blood EPA + DHA levels are independently associated with slower rates of telomere shortening,^5^ a purported marker of “cellular aging,” is yet another favorable biomarker relationship, but it is not a molecular mechanism.

From an observational study, we cannot conclude that raising the Omega-3 Index will have heart benefits and/or prolong life. Nevertheless, it is instructive to consider how much more EPA + DHA an individual might need to consume to move the median Omega-3 Index in quintile 1 (3.7%) to that in quintile 5 (7.8%). Based on a recent dose-response study,^60^ one can estimate that it would take about 1300 more mg of EPA + DHA per day to do this. This amount of EPA + DHA could be obtained from a single serving (100 g) of farmed salmon^61^ daily. Alternatively, 4 standard fish oil pills per day would suffice. Several early omega-3 RCTs used 800–1000 mg/d and reported reduced total mortality,^6,7,62^ whereas other more recent studies at the same dose have not.^9,11,63^ Several possible reasons for this discrepancy have been proposed.^64,65^

### Strengths and limitations

The strengths of our investigation include the relatively large sample size from a well-characterized community, the unambiguous nature of the primary endpoint, and the use of an objective and standardized biomarker of PUFA exposure that has low biological variability.^66^ Weaknesses include the assessment of PUFA exposure at only one point in time (which cannot capture changes in PUFA status that may have occurred during follow-up) and the largely Caucasian cohort (which precludes firm conclusions regarding other races). Also, the relatively low event rates, especially for CVD death, limited our ability to detect associations with this endpoint. Although clearly affected by omega-3 PUFA intake, other factors can influence the Omega-3 Index such as smoking and body weight,^67^ both of which were controlled for here. What we did not (and could not) control for is heritability, which we showed previously to explain about 24% of the variability in this biomarker.^67^ In the same vein, we did not include in our models the intake of key nutrients (kilocalories, saturated FAs, salt, fiber, etc.), which might contribute to the relationships reported here. However, by controlling for some of the physiological/metabolic consequences of consuming diets of poor quality (body weight, blood pressure, hyperlipidemia, and CRP levels), as well as for socioeconomic variables (which themselves track with poor diets), it is unlikely that including individual nutrients would have altered the outcome of the study. As always, the presence of residual or unmeasured confounding also precludes inferences about causality. For example, despite controlling for multiple lifestyle/health factors, higher omega-3 PUFA levels may simply be a marker for a healthier overall diet and behaviors, which themselves, independent of the omega-3 levels, may be cardioprotective.

In conclusion, we observed that higher RBC levels of n-3 PUFAs were associated with greater longevity and reduced risk for several CVD-related endpoints in this community-based sample. These findings, in the context of the totality of available evidence on this topic, provide further support for a role for the use of the Omega-3 Index in risk stratification algorithms.

## Figures and Tables

**Table 1:** Demographic overview (n = 2500)

	Died (n = 350)	Still living (n = 2150)	Total
	
Variable	% (n) or mean (SD)		
Sex			
Male	52.9% (185)	41.5% (893)	43.1% (1078)
Female	47.1% (165)	58.5% (1257)	56.9% (1422)
Age	72.9 (8.6)	64.4 (8.2)	65.56 (8.76)
BMI	27.9 (5.9)	28.3 (5.4)	28.2 (5.4)
Marital status			
Single/never married	4.3% (15)	6.7% (144)	6.4% (159)
Married	63.7% (223)	70.7% (1519)	69.7% (1742)
Separated/divorced	12.0% (42)	12.7% (273)	12.6% (315)
Widowed	18.3% (64)	9.4% (203)	10.7% (267)
Education			
Some high school or less	6.0% (21)	2.4% (51)	2.9% (72)
High school graduate	33.7% (118)	24.8% (533)	26.0% (651)
Some college or vocational	22.9% (78)	21.9% (471)	22.0% (549)
College graduate	36.6% (128)	50.5% (1086)	48.6% (1214)
Employment			
Employed	31.1% (109)	56.3% (1210)	52.8% (1319)
Disabled/unemployed	3.1% (11)	2.5% (53)	2.6% (64)
Retired	64.3% (225)	40.8% (877)	44.1% (1102)
Health insurance status			
No insurance	2.3% (8)	1.9% (40)	1.9% (48)
Insurance, but no prescription	11.4% (40)	8.4% (181)	8.8% (221)
Full insurance	84.0% (294)	88.7% (1906)	88.0% (2200)
Regular aspirin use	49.7% (174)	38.6% (830)	40.2% (1004)
Prevalent hypertension	57.1% (200)	42.2% (908)	44.3% (1108)
Cholesterol medication	41.4% (145)	36.9% (794)	37.6% (939)
Prevalent diabetes	22.3% (78)	11.6% (249)	13.1% (327)
Alcohol consumption			
None	34.0% (119)	23.2% (499)	24.7% (618)
<1 drink per day	36.0% (126)	50.7% (1089)	48.6% (1215)
1–2 drinks per day	22.6% (79)	20.2% (434)	20.5% (513)
>2 drinks per day	6.9% (24)	5.7% (123)	5.9% (147)
Smoking			
Not current smoker	89.1% (312)	90.5% (1946)	90.3% (2258)
Current	10.6% (37)	9.3% (200)	9.5% (237)
METS	4.7 (15.4)	3.3 (8.6)	3.5 (9.9)
Total to HDL cholesterol ratio	3.5 (1.1)	3.5 (1.0)	3.5 (1.1)
Systolic BP	133.6 (19.8)	128.2 (16.9)	129.0 (17.4)
C-reactive protein	4.9 (12.7)	3.0 (6.1)	3.3 (7.4)

BMI, body mass index; METS, metabolic equivalents; HDL, high-density lipoprotein; BP, blood pressure.

**Table 2:** Adjusted risk of events by Omega-3 Index (n = 2500)^§^

	Hazard ratios (95% CIs)		
	Total CVD	Total CHD	Total stroke
Omega-3 Index			
<4.2% (n = 508)	1.0	1.0	1.0
4.2%–4.9% (n = 500)	1.06 (0.69, 1.63)	1.05 (0.60, 1.83)	1.16 (0.60, 2.24)
4.9%–5.7% (n = 501)	1.10 (0.72, 1.68)	0.81 (0.44, 1.48)	1.30 (0.68, 2.49)
5.7%–6.8% (n = 502)	0.74 (0.47, 1.17)	0.63 (0.34, 1.20)	0.59 (0.28, 1.25)
>6.8% (n = 489)	***0.61 (0.37, 0.99)*** ^*^	0.58 (0.29, 1.18)	0.45 (0.20, 1.03)
*P*-value for linear trend	***.008*** ^†^	***.03*** ^*^	***.006*** ^†^

CVD, cardiovascular disease; CHD, coronary heart disease; CI, confidence interval.

* *P* < .05

† *P* < .01.

All significant hazard ratios/*P*-values are shown in bold italics.

§ Adjusted for all variables in Table 1 except baseline CVD.

**Table 3:** Adjusted risk of events and mortality by individual omega-3 fatty acids (n = 2500)^§^

	Hazard ratios (95% CIs)						
	Total Events			Mortality			
	CVD	CHD	Stroke	CVD	Cancer	Other	Total
α-linolenic acid							
<4.2% (n = 483)	1.0	1.0	1.0	1.0	1.0	1.0	1.0
4.2%–4.9% (n = 496)	***1.45 (0.94, 2.22)***	1.44 (0.78, 2.65)	1.59 (0.77, 3.25)	1.76 (0.62, 4.93)	0.71 (0.41, 1.21)	0.51 (0.26, 1.02)	0.84 (0.58, 1.21)
4.9%–5.7% (n = 506)	0.98 (0.62, 1.56)	1.00 (0.52, 1.92)	0.93 (0.43, 2.02)	1.79 (0.59, 5.44)	1.05 (0.65, 1.71)	0.92 (0.49, 1.70)	1.04 (0.72, 1.50)
5.7%–6.8% (n = 505)	1.00 (0.63, 1.61)	0.78 (0.40, 1.53)	1.27 (0.62, 2.64)	***3.06 (1.09, 8.60)*** ^*^	0.54 (0.28, 1.03)	0.84 (0.46, 1.54)	0.96 (0.67, 1.39)
>6.8% (n = 510)	1.16 (0.75, 1.80)	1.03 (0.55, 1.94)	1.42 (0.69, 2.92)	1.78 (0.62, 5.06)	0.91 (0.54, 1.53)	0.85 (0.48, 1.52)	1.04 (0.73, 1.47)
*P*-value for linear trend .	84	.43	.59	.16	.48	.94	.64
Eicosapentaenoic acid							
<0.44% (n = 494)	1.0	1.0	1.0	1.0	1.0	1.0	1.0
0.44%–0.55% (n = 495)	1.34 (0.90, 1.99)	1.13 (0.66, 1.93)	1.61 (0.83, 3.15)	0.72 (0.30, 1.73)	***0.50 (0.27, 0.93)*** ^*^	0.98 (0.54, 1.77)	0.78 (0.55, 1.10)
0.55%–0.68% (n = 510)	1.20 (0.77, 1.87)	0.74 (0.30, 1.38)	1.78 (0.88, 3.60)	0.64 (0.26, 1.62)	0.69 (0.40, 1.19)	0.81 (0.47, 1.41)	***0.66 (0.47, 0.95)*** ^*^
0.68%–0.92% (n = 508)	0.77 (0.49, 1.20)	***0.45 (0.24, 0.86)*** ^*^	1.13 (0.54, 2.35)	0.81 (0.32, 2.05)	***0.58 (0.35, 0.97)*** ^*^	***0.41 (0.22, 0.79)*** ^†^	***0.58 (0.41, 0.84)*** ^†^
>0.92% (n = 493)	0.87 (0.55, 1.39)	0.69 (0.37, 1.29)	0.95 (0.43, 2.11)	0.58 (0.21, 1.61)	0.83 (0.49, 1.41)	0.71 (0.39, 1.30)	0.74 (0.52, 1.06)
*P*-value for linear trend	.11	***.02*** ^*^	.57	.36	.63	***.03*** ^*^	***.03***
Docosapentaenoic acid							
<0.44% (n = 493)	1.0	1.0	1.0	1.0	1.0	1.0	1.0
0.44%–0.55% (n = 504)	1.17 (0.78, 1.77)	0.86 (0.49, 1.51)	2.09 (1.05, 4.16)^*^	0.64 (0.24, 1.70)	1.02 (0.60, 1.73)	1.34 (0.76, 2.34)	0.93 (0.66, 1.29)
0.55%–0.68% (n = 520)	0.83 (0.55, 1.27)	0.64 (0.36, 1.15)	1.24 (0.60, 2.54)	0.92 (0.37, 2.27)	0.52 (0.27, 0.99)^*^	0.89 (0.48, 1.67)	0.71 (0.50, 1.01)
0.68%–0.92% (n = 497)	0.87 (0.56, 1.36)	0.83 (0.47, 1.48)	1.22 (0.57, 2.63)	0.40 (0.14, 1.14)	0.86 (0.49, 1.51)	0.91 (0.45, 1.85)	0.74 (0.51, 1.07)
>0.92% (n = 486)	0.92 (0.58, 1.46)	0.60 (0.31, 1.15)	1.17 (0.52, 2.66)	0.90 (0.34, 2.37)	1.15 (0.68, 1.94)	0.60 (0.30, 1.17)	0.81 (0.56, 1.17)
*P*-value for linear trend	.31	.13	.65	.58	.87	.059	.10
Docosahexaenoic acid							
<3.69% (n = 513)	1.0	1.0	1.0	1.0	1.0	1.0	1.0
3.69%–4.36% (n = 498)	0.98 (0.64, 1.50)	0.90 (0.51, 1.57)	1.14 (0.60, 2.17)	0.67 (0.26, 1.71)	0.70 (0.41, 1.19)	***0.43 (0.24, .0.78)*** ^†^	0.78 (0.55, 1.11)
4.36%–5.01% (n = 498)	0.99 (0.65, 1.50)	0.68 (0.37, 1.25)	1.27 (0.68, 2.38)	0.61 (0.22, 1.72)	0.62 (0.35, 1.11)	***0.38 (0.20, 0.71)*** ^†^	***0.61 (0.42, 0.88)*** ^†^
5.01%–5.96% (n = 504)	0.71 (0.44, 1.12)	0.61 (0.32, 1.14)	0.57 (0.27, 1.20)	0.76 (0.29, 1.97)	0.77 (0.45, 1.31)	***0.47 (0.26, 0.87)*** ^*^	0.72 (0.50, 1.05)
>5.96% (n = 487)	***0.57 (0.35, 0.92)*** ^*^	0.54 (0.27, 1.07)	***0.41 (0.18, 0.93)*** ^*^	0.43 (0.17, 1.10)	0.92 (0.54, 1.56)	***0.47 (0.27, 0.81)*** ^†^	***0.68 (0.47, 0.99)*** ^*^
*P*-value for linear trend	***.004*** ^†^	***.03***	***.002*** ^†^	.14	.98	.054	.06

CVD, cardiovascular disease; CHD, coronary heart disease; CI, confidence interval.

* *P* < .05

† *P* < .01.

All significant hazard ratios/*P*-values are shown in bold italics.

§ Adjusted for all variables in Table 1 except history of CVD.

**Table 4:** Omega-3 Index and total cholesterol: Associations with risk for disease outcomes (n = 2500)

	Hazard ratios (95% CIs)				
Biomarker	Total CVD	Total CHD	Total Stroke	CVD mortality	Any Mortality
Omega-3 Index^§^					
<4.2% (n = 506)	1.0	1.0	1.0	1.0	1.0
4.2%–4.9% (n = 500)	1.08 (0.70, 1.65)	1.06 (0.61, 1.85)	1.20 (0.63, 2.27)	0.65 (0.27, 1.54)	0.74 (0.53, 1.03)
4.9%–5.7% (n = 500)	1.11 (0.73, 1.68)	0.81 (0.44, 1.47)	1.32 (0.69, 2.50)	0.53 (0.19, 1.49)	***0.67 (0.47, 0.97)***
5.7%–6.8% (n = 502)	0.74 (0.47, 1.17)	0.63 (0.34, 1.19)	0.61 (0.29, 1.27)	0.58 (0.22, 1.55)	***0.58 (0.41, 0.84)*** ^†^
>6.8% (n = 489)	0.63 (0.39, 1.01)	0.59 (0.30, 1.17)	0.47 (0.21, 1.06)	0.44 (0.16, 1.91)	***0.65 (0.45, 0.94)***
*P*-value from linear trend test^ǁ^	***.009*** ^†^	***.03*** ^*^	***.006*** ^†^	.19	***.01*** ^*^
Total cholesterol^^§^^					
<154 (n = 406)	1.0	1.00	1.0	1.0	1.0
154–175 (n = 491)	1.03 (0.69, 1.56)	1.02 (0.55, 1.89)	0.88 (0.47, 1.66)	1.22 (0.53, 2.77)	0.73 (0.50, 1.05)
176–194 (n = 520)	0.95 (0.62, 1.45)	1.29 (0.71, 2.37)	0.63 (0.31, 1.27)	0.67 (0.26, 1.77)	0.72 (0.49, 1.06)
195–218 (n = 551)	0.89 (0.56, 1.39)	1.01 (0.53, 1.92)	0.69 (0.32, 1.40)	1.07 (0.30, 3.79)	0.91 (0.64, 1.31)
>218 (n = 530)	1.09 (0.66, 1.80)	1.59 (0.81, 3.11)	0.89 (0.41, 1.93)	0.31 (0.72, 1.34)	0.96 (0.66, 1.40)
*P*-value from linear trend test^ǁ^	.99	.26	.50	.27	.11

CVD, cardiovascular disease; CHD, coronary heart disease; CI, confidence interval.

* *P* < .05

† *P* < .01

All significant hazard ratios/*P*-values are shown in bold italics.

§ Hazard ratios presented here were adjusted for all variables in Table 1 with the addition of grouped total cholesterol (and removing total cholesterol to high-density lipoprotein cholesterol ratio) and the grouped Omega-3 Index.

ǁ Linear trend test models were fit for both the Omega-3 Index and TC simultaneously, after adjusting for variables as described in footnote “^§^”.

## APPENDIX

**Table S1:** Distribution of outcomes (n = 2500)

	Number of people with events (cases)	Number of people without events (controls)	Median follow-up days^*^	Maximum follow-up days^*^
Total CVD	245	2255	2351	3833
Total CHD	119	2381	2342	3815
Ischemic stroke	105	2395	2342	3833
CVD mortality	58	2442	2673	3815
Death from any cause^†^	350	2150	2686	3815

CVD, cardiovascular disease; CHD, coronary heart disease; CI, confidence interval.

* Days to event or days to censoring.

† The causes of the 350 observed deaths, besides CVD, were 146 cancer (42%), 128 other (37%), and 18 unknown (5%).

**Table S2:** Risk of fatal events by Omega-3 Index (n = 2500)

	Hazard ratios (95% CIs)			
	CVD mortality	Cancer mortality	Other mortality	Any mortality
Omega-3 Index–unadjusted				
<4.2% (n = 508)	1.0	1.0	1.0	1.0
4.2%–4.9% (n = 500)	0.97 (0.41, 2.30)	0.94 (0.58, 1.55)	0.59 (0.34, 1.02)	0.81 (0.59, 1.12)
4.9%–5.7% (n = 501)	0.96 (0.43, 2.16)	0.88 (0.50, 1.57)	0.63 (0.36, 1.09)	0.78 (0.56, 1.10)
5.7%–6.8% (n = 502)	1.10 (0.46, 2.62)	0.72 (0.42, 1.23)	0.67 (0.40, 1.11)	***0.73 (0.53, 1.02)***
>6.8% (n = 489)	0.88 (0.39, 2.01)	1.19 (0.72, 1.97)	***0.53 (0.31, 0.91)*** ^*^	0.81 (0.58, 1.12)
*P*-value for linear trend	.86	.85	.07	.16
Omega-3 Index–adjusted^§^				
<4.2% (n = 508)	1.0	1.0	1.0	1.0
4.2%–4.9% (n = 500)	0.57 (0.23, 1.40)	0.66 (0.38, 1.14)	***0.54 (0.31, 0.94)*** ^*^	0.74 (0.53, 1.04)
4.9%–5.7% (n = 501)	0.54 (0.20, 1.47)	0.73 (0.42, 1.28)	***0.38 (0.20, 0.73)*** ^†^	***0.67 (0.46, 0.96)*** ^*^
5.7%–6.8% (n = 502)	0.56 (0.22, 1.43)	0.60 (0.35, 1.03)	***0.44 (0.25, 0.79)*** ^†^	***0.59 (0.41, 0.86)*** ^†^
>6.8% (n = 489)	0.39 (0.15, 1.02)	0.96 (0.56, 1.64)	***0.44 (0.24, 0.79)*** ^†^	***0.66 (0.45, 0.96)*** ^*^
*P*-value for linear trend	.10	.88	***.009*** ^†^	***.02*** ^*^

CVD, cardiovascular disease; CI, confidence interval.

* P < .05

† *P* < .01

‡ *P* < .001.

All significant hazard ratios/*P*-values are shown in bold italics.

§ Adjusted for all variables in Table 1 except baseline CVD.

**Table S3:** Risk of events by Omega-3 Index modified to include docosapentaenoic acid (n = 2500)

	Hazard ratios (95% CIs			
	Total CVD	Total CHD	Total stroke	Any mortality
Omega-3 Index–adjusted				
<4.2% (n = 508)	1.0	1.0	1.0	1.0
4.2%–4.9% (n = 500)	1.06 (0.69, 1.63)	1.05 (0.60, 1.83)	1.16 (0.60, 2.24)	0.74 (0.53, 1.04)
4.9%–5.7% (n = 501)	1.10 (0.72, 1.68)	0.81 (0.44, 1.48)	1.30 (0.68, 2.49)	***0.67 (0.46, 0.96)*** ^*^
5.7%–6.8% (n = 502)	0.74 (0.47, 1.17)	0.63 (0.34, 1.20)	0.59 (0.28, 1.25)	***0.59 (0.41, 0.86)*** ^†^
>6.8% (n = 489)	***0.61 (0.37, 0.99)*** ^*^	0.58 (0.29, 1.18)	0.45 (0.20, 1.03)	***0.66 (0.45, 0.96)*** ^*^
*P*-value for linear trend	***.008*** ^†^	***.03*** ^*^	***.006*** ^†^	***.02*** ^*^
Modified Omega-3 Index (+DPA)–adjusted				
<6.7% (n = 510)	1.0	1.0	1.0	1.0
6.7%–7.5% (n = 497)	1.01 (0.66, 1.54)	0.93 (0.54, 1.61)	1.04 (0.52, 2.07)	0.75 (0.53, 1.06)
7.5%–8.4% (n = 502)	0.83 (0.54, 1.27)	0.58 (0.32, 1.05)	1.09 (0.57, 2.08)	***0.48 (0.40, 0.84)*** ^†^
8.4%–9.7% (n = 507)	0.69 (0.44, 1.08)	0.54 (0.29, 1.01)	0.65 (0.31, 1.35)	***0.72 (0.50, 1.04)***
>9.7% (n = 484)	0.64 (0.40, 1.03)	0.54 (0.27, 1.05)	0.51 (0.23, 1.14)	***0.61 (0.41, 0.90)*** ^*^
*P*-value for linear trend	***.012*** ^*^	***.013*** ^*^	***.024*** ^*^	***.02*** ^*^

CVD, cardiovascular disease; CHD, coronary heart disease; CI, confidence interval.

* *P* < .05

† *P* < .01

‡ *P* < .001.

All significant hazard ratios/*P*-values are shown in bold italics.

Adjusted for all variables in Table 1.

**Table S4:** Risk of events by Omega-3 Index risk categories (n = 2500)

	Hazard ratios (95% CIs)			
	Total CVD	Total CHD	Total stroke	Any mortality
Omega-3 Index–unadjusted				
<4% (n = 416)	1.0	1.0	1.0	1.0
4%–8% (n = 1876)	1.03 (0.72, 1.48)	0.94 (0.57, 1.54)	1.12 (0.64, 1.94)	***0.73 (0.56, 0.96)*** ^*^
>8% (n = 208)	0.77 (0.43, 1.38)	0.73 (0.34, 1.58)	0.88 (0.33, 2.32)	0.85 (0.68, 1.07)
*P*-value for linear trend	.45	.48	.93	.15
Omega-3 Index–adjusted^§^				
<4% (n = 416)	1.0	1.0	1.0	1.0
4%–8% (n = 1876)	0.88 (0.59, 1.32)	0.85 (0.49, 1.47)	0.91 (0.48, 1.71)	***0.61 (0.45, 0.83)*** ^†^
>8% (n = 208)	0.68 (0.36, 1.27)	0.83 (0.35, 1.94)	0.50 (0.17, 1.52)	0.69 (0.43, 1.13)
*P*-value for linear trend	.59	.23	.21	***.04*** ^*^

CVD, cardiovascular disease; CHD, coronary heart disease; CI, confidence interval.

* *P* < .05

† *P* < .01

‡ *P* < .001.

All significant hazard ratios/*P*-values are shown in bold italics.

§ Adjusted for all variables in Table 1.

**Table S5:** The Omega-3 Index vs the n-6:n-3 ratio and vs the Omega-3 Index adjusting for all n-6 fatty acids (n = 2500)

	Hazard ratios (95% CIs)						
	Total CVD	Total CHD	Total stroke	CVD mortality	Cancer mortality	Other mortality	Any Mortality
Omega-3 Index ^§^							
<4.2%	1.0	1.0	1.0	1.0	1.0	1.0	1.0
4.2%–4.9%	1.06 (0.69, 1.63)	1.05 (0.60, 1.83)	1.16 (0.60, 2.24)	0.57 (0.23, 1.40)	0.66 (0.38, 1.14)	***0.54 (0.31, 0.94)*** ^*^	0.74 (0.53, 1.04)
4.9%–5.7%	1.10 (0.72, 1.68)	0.81 (0.44, 1.48)	1.30 (0.68, 2.49)	0.54 (0.20, 1.47)	0.73 (0.42, 1.28)	***0.38 (0.20, 0.73)*** ^†^	***0.67 (0.46, 0.96)*** ^*^
5.7%–6.8%	0.74 (0.47, 1.17)	0.63 (0.34, 1.20)	0.59 (0.28, 1.25)	0.56 (0.22, 1.43)	0.60 (0.35, 1.03)	***0.44 (0.25, 0.79)*** ^†^	***0.59 (0.41, 0.86)*** ^†^
>6.8%	***0.61 (0.37, 0.99)*** ^*^	0.58 (0.29, 1.18)	0.45 (0.20, 1.03)	0.39 (0.15, 1.02)	0.96 (0.56, 1.64)	***0.44 (0.24, 0.79)*** ^†^	***0.66 (0.45, 0.96)*** ^*^
*P*-value for trend	***.008*** ^†^	***.03*** ^*^	***.006*** ^†^	.10	.88	***.009*** ^†^	***.02*** ^*^
n6:n3 ratio^§^							
<3.31	1.0	1.0	1.0	1.0	1.0	1.0	1.0
3.32–3.99	1.11 (0.70, 1.78)	0.93 (0.33, 1.96)	1.34 (0.56, 3.19)	1.17 (0.45, 3.02)	0.65 (0.37, 1.15)	1.20 (0.66, 2.20)	0.98 (0.67, 1.41)
3.99–4.56	***1.69 (1.10, 2.62)*** ^*^	1.26 (0.65, 2.46)	***3.08 (1.45, 6.54)*** ^†^	1.41 (0.58, 3.46)	0.83 (0.48, 1.42)	0.83 (0.43, 1.59)	1.00 (0.69, 1.45)
4.56–5.20	***1.64 (1.06, 2.53)*** ^*^	1.62 (0.86, 3.06)	***2.52 (1.15, 5.54)*** ^*^	1.12 (0.45, 2.75)	0.75 (0.42, 1.35)	1.12 (0.62, 2.00)	1.11 (0.78, 1.59)
>5.20	1.61 (0.99, 2.62)	1.88 (0.95, 3.74)	2.02 (0.88, 4.65)	2.46 (0.93, 6.51)	1.12 (0.65, 1.93)	***2.43 (1.36, 4.35)*** ^†^	***1.58 (1.08, 2.31)*** ^*^
*P*-value for trend	***.008*** ^†^	***.014*** ^*^	***.012*** ^*^	.14	.68	***.023*** ^†^	***.019***^*^
Omega-3 Index + all n-6 fatty acids^§^							
<4.2%	1.0	1.0	1.0	1.0	1.0	1.0	1.0
4.2%–4.9%	1.03 (0.67, 1.57)	1.04 (0.59, 1.83)	1.15 (0.59, 2.24)	0.52 (0.20, 1.38)	0.66 (0.38, 1.14)	0.43 (0.23, 0.78)	0.67 (0.47, 0.95)^*^
4.9%–5.7%	1.04 (0.67, 1.62)	0.85 (0.45, 1.58)	1.22 (0.60, 2.47)	0.46 (0.16, 1.35)	0.72 (0.39, 1.32)	***0.32 (0.17, 0.62)*** ^‡^	***0.63 (0.43, 0.92)*** ^*^
5.7%–6.8%	0.65 (0.40, 1.08)	0.65 (0.32, 1.34)	0.50 (0.22, 1.15)	0.52 (0.18, 1.46)	0.55 (0.28, 1.10)	***0.33 (0.16, 0.68)*** ^†^	***0.52 (0.34, 0.80)*** ^†^
>6.8%	***0.48 (0.26, 0.91)*** ^*^	0.62 (0.26, 1.44)	0.33 (0.10, 1.04)	0.37 (0.10, 1.31)	0.92 (0.41, 2.10)	***0.32 (0.13, 0.78)*** ^*^	0.61 (0.36, 1.04)
*P*-value from trend	***.014***	.14	***.02*** ^*^	.16	.58	***.006*** ^†^	***.025*** ^*^

CVD, cardiovascular disease; CHD, coronary heart disease; CI, confidence interval.

* *P* < .05

† *P* < .01

‡ *P* < .001.

All significant hazard ratios/*P*-values are shown in bold italics.

§ Adjusted for all variables in Table 1 except baseline CVD.

**Table S6:** Risk of fatal events by Omega-3 Index (n = 2899)

	Hazard ratios (95% CIs)			
	Cancer mortality	Other mortality	CVD mortality	Any mortality
Omega-3 Index–unadjusted				
<4.2% (n = 580)	1.0	1.0	1.0	1.0
4.2%–4.9% (n = 579)	0.88 (0.58, 1.33)	0.66 (0.41, 1.07)	0.84 (0.44, 1.61)	0.83 (0.63, 1.09)
4.9%–5.7% (n = 580)	0.86 (0.53, 1.38)	0.62 (0.39, 1.01)	0.93 (0.50, 1.73)	0.78 (0.58, 1.04)
5.7%–6.8% (n = 580)	0.61 (0.37, 1.00)	0.65 (0.41, 1.03)	1.06 (0.56, 2.00)	***0.72 (0.54, 0.96)*** ^*^
>6.8% (n = 480)	0.97 (0.62, 1.51)	***0.58 (0.37, 0.92)*** ^*^	0.92 (0.51, 1.68)	0.79 (0.60, 1.04)
*P*-value for linear trend	.51	***.046*** ^*^	.96	.05
Omega-3 Index–adjusted				
<4.2% (n = 580)	1.0	1.0	1.0	1.0
4.2%–4.9% (n = 579)	0.70 (0.43, 1.12)	***0.65 (0.41, 1.03)***	0.57 (0.23, 1.42)	0.79 (0.59, 1.06)
4.9%–5.7% (n = 580)	0.75 (0.46, 1.21)	***0.38 (0.22, 0.67)*** ^‡^	0.90 (0.45, 1.76)	***0.70 (0.52, 0.95)*** ^*^
5.7%–6.8% (n = 580)	***0.55 (0.33, 0.91)*** ^*^	***0.52 (0.31, 0.87)*** ^*^	0.88 (0.42, 1.85)	***0.66 (0.49, 0.90)*** ^†^
>6.8% (n = 480)	0.87 (0.55, 1.39)	***0.49 (0.29, 0.84)*** ^†^	0.74 (0.36, 1.50)	***0.71 (0.52, 0.97)*** ^*^
*P*-value for linear trend	.45	***.01*** ^*^	.87	***.02*** ^*^

CVD, cardiovascular disease; CHD, coronary heart disease; CI, confidence interval.

* *P* < .05

† *P* < .01

‡ *P* < .001.

All significant hazard ratios/*P*-values are shown in bold italics.

Adjusted for all variables in Table 1 except baseline CVD.

**Table S7:** Unadjusted risk of events by Omega-3 Index (n = 2500)

	Hazard ratios (95% CIs)		
	Total CVD	Total CHD	Total stroke
Omega-3 Index–unadjusted			
<4.2% (n = 508)	1.0	1.0	1.0
4.2%–4.9% (n = 500)	1.08 (0.72, 1.63)	1.07 (0.63, 1.82)	1.19 (0.65, 2.20)
4.9%–5.7% (n = 501)	1.33 (0.90, 1.96)	0.88 (0.50, 1.53)	***1.64 (0.92, 2.93)***
5.7%–6.8% (n = 502)	0.88 (0.57, 1.34)	0.73 (0.41, 1.33)	0.77 (0.39, 1.53)
>6.8% (n = 489)	0.70 (0.45, 1.10)	0.59 (0.32, 1.07)	0.68 (0.32, 1.45)
*P*-value for linear trend	.07	***.03*** ^*^	.16

CVD, cardiovascular disease; CHD, coronary heart disease; CI, confidence interval.

* *P* < .05

† *P* < .01

‡ *P* < .001.

All significant hazard ratios/*P*-values are shown in bold italics.

**Table S8:** Risk of CVD-related events by Omega-3 Index (n = 2899)

	Hazard ratios (95% CIs)		
	Total CVD	Total CHD	Total stroke
Omega-3 Index–unadjusted			
<4.2% (n = 580)	1.0	1.0	1.0
4.2%–4.9% (n = 579)	1.07 (0.76, 1.52)	0.81 (0.51, 1.28)	1.53 (0.88, 2.66)
4.9%–5.7% (n = 580)	1.24 (0.89, 1.73)	0.74 (0.47, 1.18)	***1.77 (1.03, 3.02)*** ^*^
5.7%–6.8% (n = 580)	0.99 (0.70, 1.41)	0.80 (0.51, 1.27)	1.20 (0.67, 2.14)
>6.8% (n = 580)	0.88 (0.62, 1.26)	0.65 (0.41, 1.04)	1.19 (0.65, 2.20)
*P*-value for linear trend	.40	.10	.92
Omega-3 Index–adjusted^§^			
<4.2% (n = 580)	1.0	1.0	1.0^ǁ^
4.2%–4.9% (n = 579)	1.11 (0.74, 1.67)	1.08 (0.67, 1.74)	1.36 (0.77, 2.45)
4.9%–5.7% (n = 580)	1.31 (0.90, 1.93)	0.85 (0.50, 1.44)	1.20 (0.63, 2.28)
5.7%–6.8% (n = 580)	1.00 (0.68, 1.46)	0.73 (0.46, 1.17)	0.76 (0.41, 1.40)
>6.8% (n = 580)	0.82 (0.54, 1.25)	***0.56 (0.32, 0.99)*** ^*^	0.51 (0.26, 1.02)
*P*-value for linear trend	.25	***.01*** ^*^	***.003***

CVD, cardiovascular disease; CHD, coronary heart disease; CI, confidence interval.

* *P* < .05

† *P* < .01

‡ *P* < .001.

All significant hazard ratios/*P*-values are shown in bold it.

§ Adjusted for all variables in Table 1.

ǁ The highest quintile of the Omega-3 Index had significantly lower risk for total stroke than the second quintile [0.32 (0.15, 0.68)^†^] and the third quintile [0.38 (0.19, 0.74)^†^] after adjusted for all variables in Table 1.

**Table S9:** Unadjusted risk of events and mortality by individual omega-3 fatty acids (n = 2500)

	Hazard ratios (95% CIs)						
	Total Events			Mortality			
	CVD	CHD	Stroke	CVD	Cancer	Other	Total
α-linolenic acid							
<4.2% (n = 483)	1.0	1.0	1.0	1.0	1.0	1.0	1.0
4.2–4.9% (n = 496)	1.36 (0.91, 2.03)	1.33 (0.77, 2.32)	1.69 (0.88, 3.26)	1.81 (0.72, 4.56)	0.76 (0.46, 1.28)	***0.52 (0.28, 0.97)*** ^*^	***0.77 (0.54, 1.09)***
4.9–5.7% (n = 506)	0.92 (0.60, 1.43)	0.88 (0.48, 1.61)	0.99 (0.48, 2.04)	1.59 (0.49, 4.26)	0.99 (0.60, 1.62)	0.81 (0.48, 1.36)	0.95 (0.68, 1.32)
5.7–6.8% (n = 505)	0.89 (0.57, 1.38)	0.73 (0.40, 1.36)	1.24 (0.62, 2.47)	1.99 (0.75, 5.27)	0.58 (0.32, 1.04)	0.78 (0.46, 1.34)	0.84 (0.60, 1.19)
>6.8% (n = 510)	1.26 (0.84, 1.90)	1.10 (0.61, 1.97)	1.68 (0.88, 3.21)	1.32 (0.52, 3.35)	0.95 (0.58, 1.57)	0.97 (0.57, 1.65)	1.04 (0.75, 1.45)
*P*-value for linear trend	.26	.51	.34	.53	.52	.81	.73
Eicosapentaenoic acid							
<0.44% (n = 494)	1.0	1.0	1.0	1.0	1.0	1.0	1.0
0.44–0.55% (n = 495)	1.32 (0.90, 1.94)	1.04 (0.62, 1.74)	1.67 (0.91, 3.07)	1.09 (0.52, 2.29)	0.64 (0.37, 1.10)	1.12 (0.66, 1.91)	0.89 (0.63, 1.22)
0.55–0.68% (n = 510)	1.07 (0.69, 1.64)	0.62 (0.34, 1.12)	1.71 (0.86, 3.38)	***0.70 (0.27, 1.81)***	0.74 (0.44, 1.27)	0.91 (0.53, 1.56)	0.74 (0.52, 1.05)
0.68–0.92% (n = 508)	0.85 (0.57, 1.28)	***0.53 (0.29, 0.95)*** ^*^	1.22 (0.64, 2.33)	1.02 (0.47, 2.23)	0.65 (0.40, 1.06)	***0.54 (0.30, 0.97)*** ^*^	***0.64 (0.46, 0.89)*** ^†^
>0.92% (n = 493)	0.78 (0.50, 1.21)	***0.57 (0.32, 1.00)*** ^*^	1.08 (0.52, 2.23)	0.72 (0.30, 1.74)	0.93 (0.56, 1.53)	0.62 (0.34, 1.12)	0.77 (0.55, 1.08)
*P*-value for linear trend	***.048*** ^*^	***.005*** ^†^	.84	.45	.68	***.009*** ^†^	***.02***
Docosapentaenoic acid							
<0.44% (n = 493)	1.0	1.0	1.0	1.0	1.0	1.0	1.0
0.44–0.55% (n = 504)	1.24 (0.84, 1.84)	0.88 (0.51, 1.51)	***2.10 (1.12, 3.96)*** ^*^	0.90 (0.43, 1.88)	1.06 (0.62, 1.81)	1.36 (0.81, 2.28)	1.06 (0.77, 1.46)
0.55–0.68% (n = 520)	0.95 (0.63, 1.44)	0.73 (0.41, 1.28)	1.37 (0.71, 2.63)	0.71 (0.33, 1.55)	0.65 (0.37, 1.15)	0.82 (0.46, 1.46)	0.72 (0.51, 1.03)
0.68–0.92% (n = 497)	0.99 (0.66, 1.49)	0.81 (0.46, 1.44)	1.48 (0.79, 2.78)	0.59 (0.24, 1.44)	0.99 (0.59, 1.66)	0.73 (0.39, 1.35)	0.83 (0.59, 1.18)
>0.92% (n = 486)	0.90 (0.58, 1.40)	0.60 (0.33, 1.09)	1.21 (0.56, 2.60)	0.78 (0.32, 1.89)	1.33 (0.80, 2.20)	0.62 (0.34, 1.12)	0.87 (0.62, 1.23)
*P*-value for linear trend	.36	.10	.98	.35	.43	***.01*** ^*^	.15
Docosahexaenoic acid							
<3.69% (n = 513)	1.0	1.0	1.0	1.0	1.0	1.0	1.0
3.69–4.36% (n = 498)	1.07 (0.72, 1.60)	1.0 (0.59, 1.70)	1.26 (0.68, 2.32)	1.09 (0.45, 2.60)	0.98 (0.60, 1.61)	***0.50 (0.28, 0.88)*** ^*^	0.84 (0.61, 1.16)
4.36–5.01% (n = 498)	1.23 (0.83, 1.82)	0.81 (0.46, 1.43)	1.53 (0.86, 2.75)	1.05 (0.46, 2.38)	0.73 (0.41, 1.32)	***0.53 (0.31, 0.93)*** ^*^	***0.69 (0.48, 0.97)*** ^*^
5.01–5.96% (n = 504)	0.86 (0.56, 1.31)	0.71 (0.40, 1.28)	0.78 (0.39, 1.55)	1.28 (0.53, 3.11)	0.88 (0.52, 1.48)	0.68 (0.41, 1.13)	0.84 (0.60, 1.16)
>5.96% (n = 487)	0.72 (0.46, 1.12)	0.59 (0.33, 1.07)	0.67 (0.31, 1.42)	1.03 (0.46, 2.34)	1.16 (0.70, 1.92)	***0.70 (0.36, 0.99)*** ^*^	0.86 (0.62, 1.19)
*P*-value for linear trend	.07	***.03***	.13	.93	.77	.25	.41

CVD, cardiovascular disease; CHD, coronary heart disease; CI, confidence interval.

All significant hazard ratios/*P*-values are shown in bold italics.

* *P* < .05

† *P* < .01

‡ *P* < .001.
